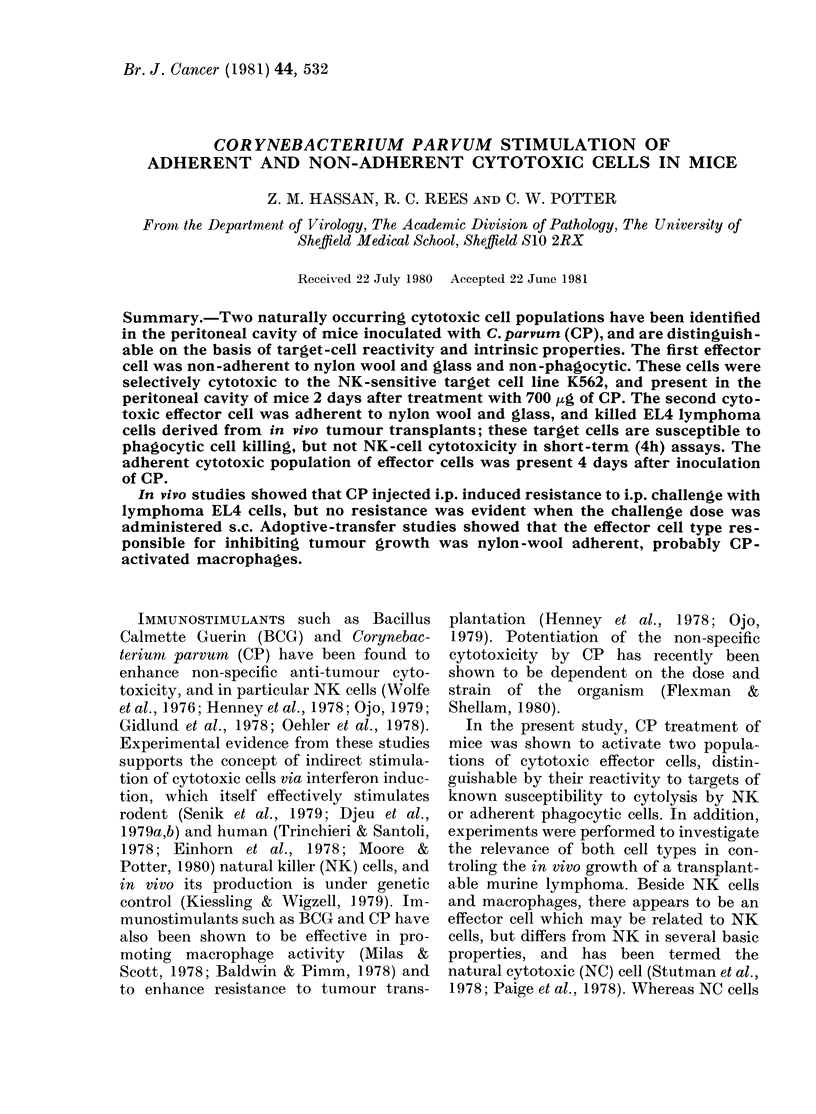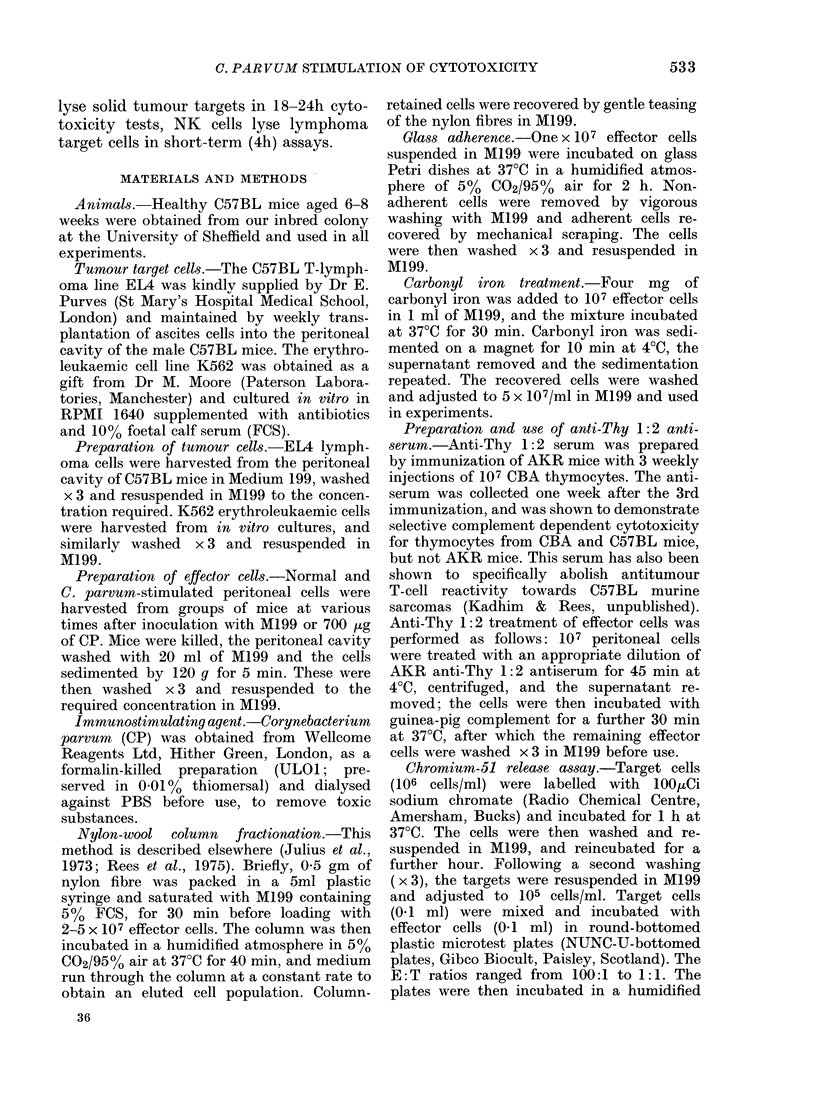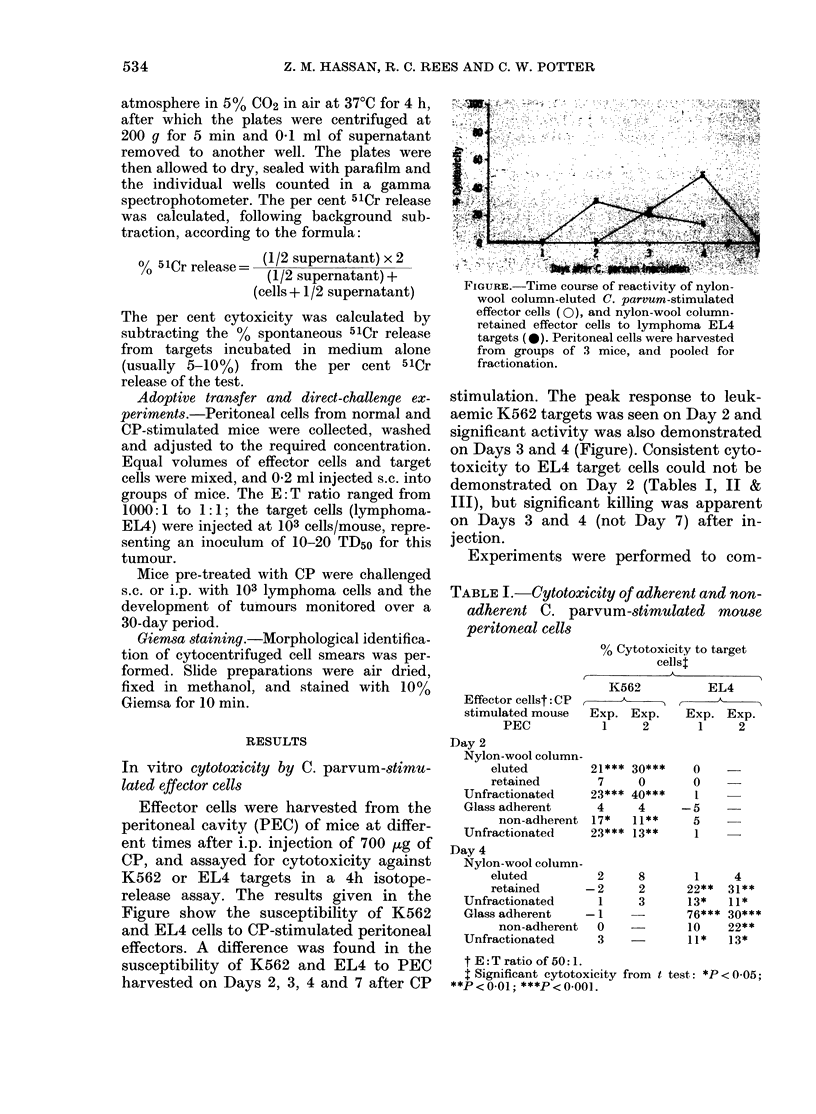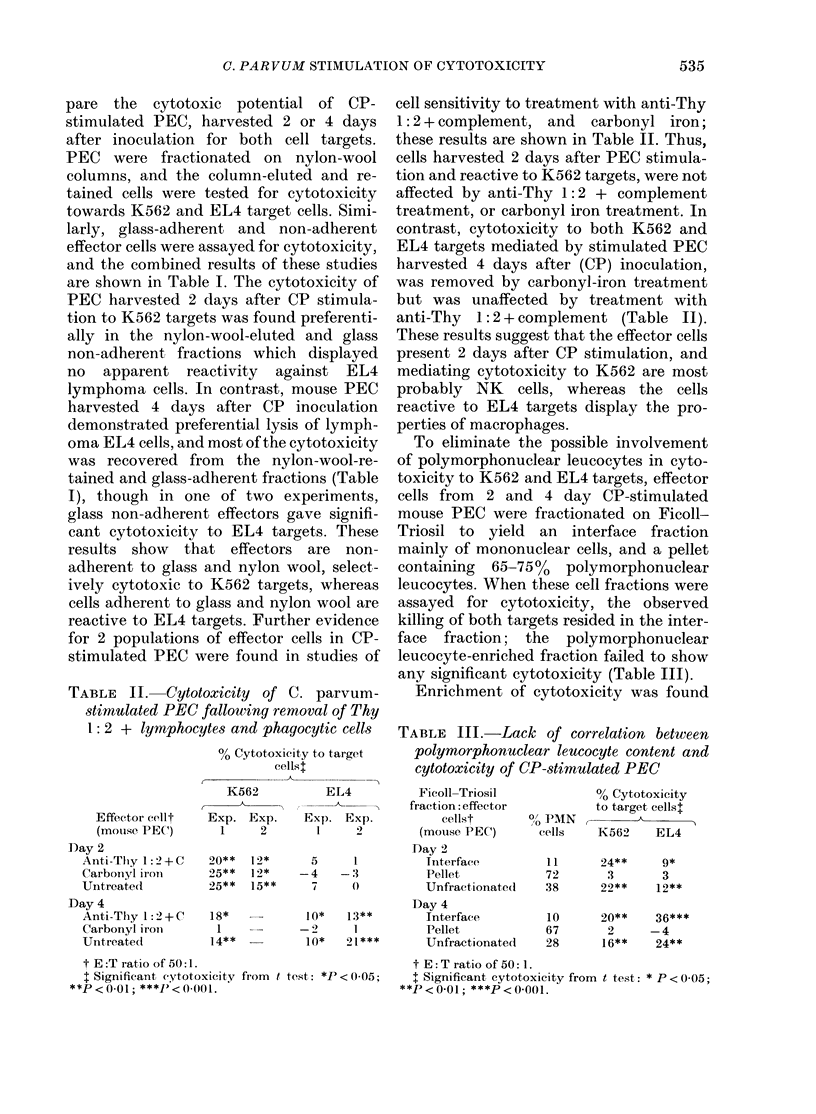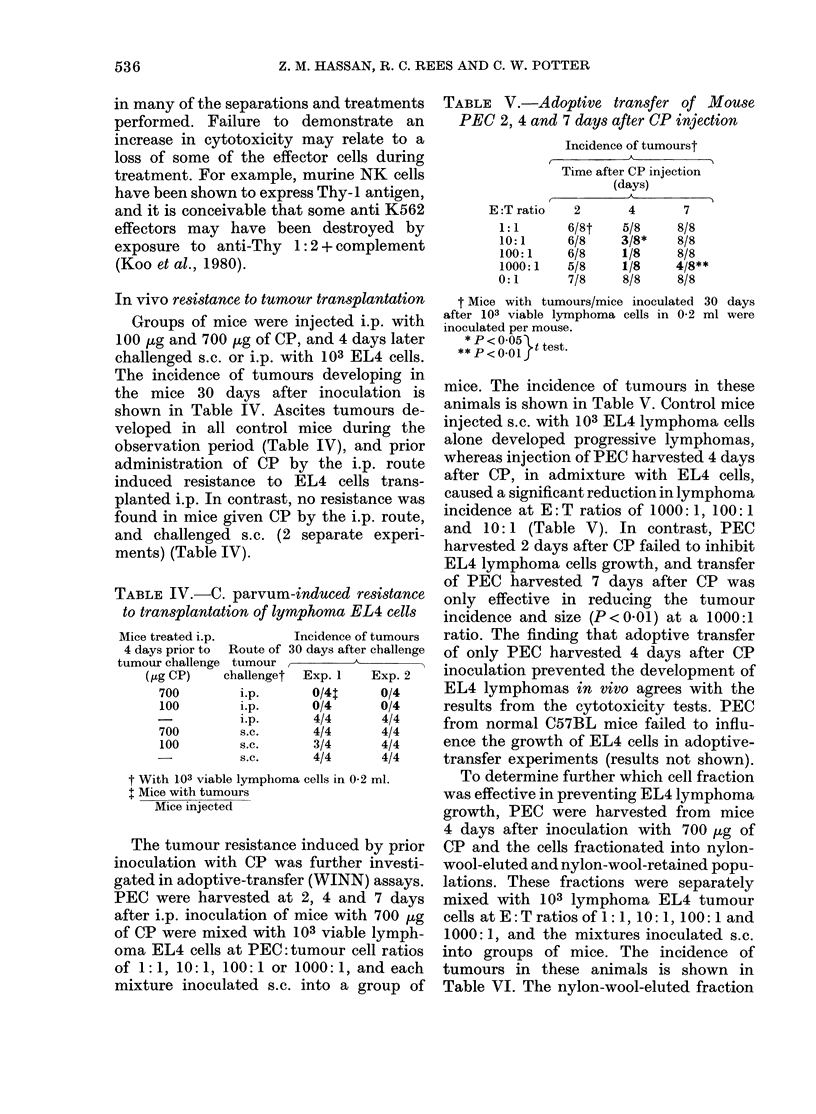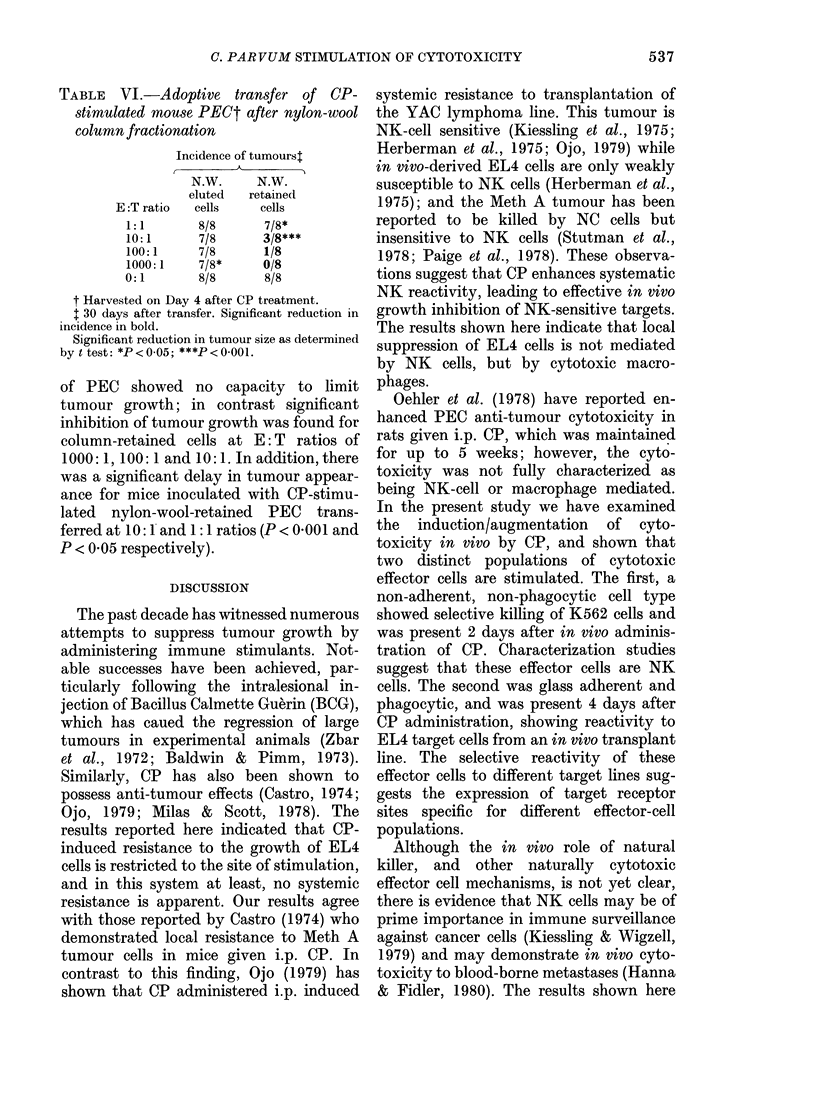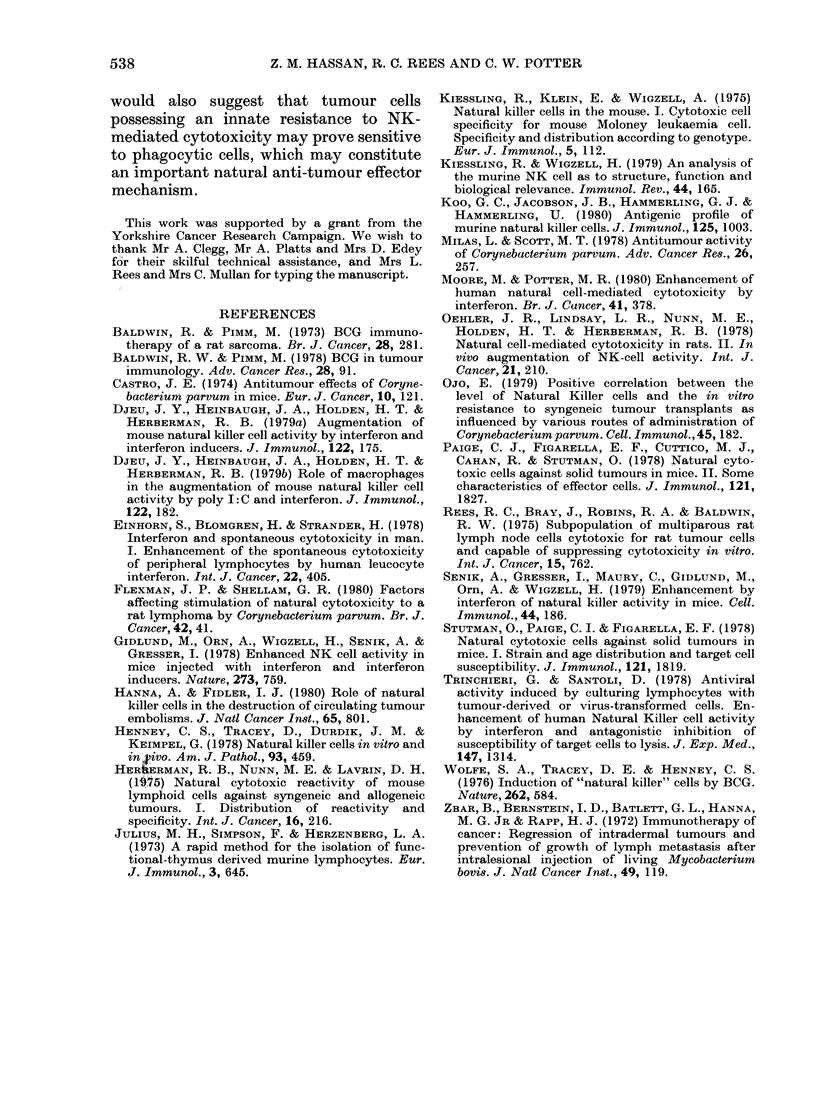# Corynebacterium parvum stimulation of adherent and non-adherent cytotoxic cells in mice.

**DOI:** 10.1038/bjc.1981.222

**Published:** 1981-10

**Authors:** Z. M. Hassan, R. C. Rees, C. W. Potter

## Abstract

Two naturally occurring cytotoxic cell populations have been identified in the peritoneal cavity of mice inoculated with C. parvum (CP), and are distinguishable on the basis of target-cell reactivity and intrinsic properties. The first effector cell was non-adherent to nylon wool and glass and non-phagocytic. These cells were selectively cytotoxic to the NK-sensitive target cell line K562, and present in the peritoneal cavity of mice 2 days after treatment with 700 micrograms of CP. The second cytotoxic effector cell was adherent to nylon wool and glass, and killed EL4 lymphoma cells derived from in vivo tumour transplants; these target cells are susceptible to phagocytic cell killing, but not NK-cell cytotoxicity in short-term (4h) assays. The adherent cytotoxic population of effector cells was present 4 days after inoculation of CP. In vivo studies showed that CP injected i.p. induced resistance to i.p. challenge with lymphoma EL4 cells, but no resistance was evident when the challenge dose was administered s.c. Adoptive-transfer studies showed that the effector cell type responsible for inhibiting tumour growth was nylon-wool adherent, probably CP-activated macrophages.


*Br. J. Cancer* (1981) **44**, 532

### *CORYNEBACTERIUM PARVUM* STIMULATION OF ADHERENT AND NON-ADHERENT CYTOTOXIC CELLS IN MICE

Z. M. HASSAN, R. C. REES AND C. W. POTTER

*From the Department of Virology, The Academic Division of Pathology, The University of Sheffield Medical School, Sheffield S10 2RX*

Received 22 July 1980 Accepted 22 June 1981

**Summary.—Two naturally occurring cytotoxic cell populations have been identified in the peritoneal cavity of mice inoculated with *C. parvum* (CP), and are distinguishable on the basis of target-cell reactivity and intrinsic properties. The first effector cell was non-adherent to nylon wool and glass and non-phagocytic. These cells were selectively cytotoxic to the NK-sensitive target cell line K562, and present in the peritoneal cavity of mice 2 days after treatment with 700 μg of CP. The second cytotoxic effector cell was adherent to nylon wool and glass, and killed EL4 lymphoma cells derived from *in vivo* tumour transplants; these target cells are susceptible to phagocytic cell killing, but not NK-cell cytotoxicity in short-term (4h) assays. The adherent cytotoxic population of effector cells was present 4 days after inoculation of CP.**

***In vivo* studies showed that CP injected i.p. induced resistance to i.p. challenge with lymphoma EL4 cells, but no resistance was evident when the challenge dose was administered s.c. Adoptive-transfer studies showed that the effector cell type responsible for inhibiting tumour growth was nylon-wool adherent, probably CP-activated macrophages.**

IMMUNOSTIMULANTS such as Bacillus Calmette Guerin (BCG) and *Corynebacterium parvum* (CP) have been found to enhance non-specific anti-tumour cytotoxicity, and in particular NK cells (Wolfe *et al.*, 1976; Henney *et al.*, 1978; Ojo, 1979; Gidlund *et al.*, 1978; Oehler *et al.*, 1978). Experimental evidence from these studies supports the concept of indirect stimulation of cytotoxic cells *via* interferon induction, which itself effectively stimulates rodent (Senik *et al.*, 1979; Djeu *et al.*, 1979*a,b*) and human (Trinchieri & Santoli, 1978; Einhorn *et al.*, 1978; Moore & Potter, 1980) natural killer (NK) cells, and *in vivo* its production is under genetic control (Kiessling & Wigzell, 1979). Immunostimulants such as BCG and CP have also been shown to be effective in promoting macrophage activity (Milas & Scott, 1978; Baldwin & Pimm, 1978) and to enhance resistance to tumour transplantation (Henney *et al.*, 1978; Ojo, 1979). Potentiation of the non-specific cytotoxicity by CP has recently been shown to be dependent on the dose and strain of the organism (Flexman & Shellam, 1980).

In the present study, CP treatment of mice was shown to activate two populations of cytotoxic effector cells, distinguishable by their reactivity to targets of known susceptibility to cytolysis by NK or adherent phagocytic cells. In addition, experiments were performed to investigate the relevance of both cell types in controling the *in vivo* growth of a transplantable murine lymphoma. Beside NK cells and macrophages, there appears to be an effector cell which may be related to NK cells, but differs from NK in several basic properties, and has been termed the natural cytotoxic (NC) cell (Stutman *et al.*, 1978; Paige *et al.*, 1978). Whereas NC cells lyse solid tumour targets in 18–24h cytotoxicity tests, NK cells lyse lymphoma target cells in short-term (4h) assays.

### MATERIALS AND METHODS

*Animals.*—Healthy C57BL mice aged 6–8 weeks were obtained from our inbred colony at the University of Sheffield and used in all experiments.

*Tumour target cells.*—The C57BL T-lymphoma line EL4 was kindly supplied by Dr E. Purves (St Mary's Hospital Medical School, London) and maintained by weekly transplantation of ascites cells into the peritoneal cavity of the male C57BL mice. The erythroleukaemic cell line K562 was obtained as a gift from Dr M. Moore (Paterson Laboratories, Manchester) and cultured *in vitro* in RPMI 1640 supplemented with antibiotics and 10% foetal calf serum (FCS).

*Preparation of tumour cells.*—EL4 lymphoma cells were harvested from the peritoneal cavity of C57BL mice in Medium 199, washed ×3 and resuspended in M199 to the concentration required. K562 erythroleukaemic cells were harvested from *in vitro* cultures, and similarly washed ×3 and resuspended in M199.

*Preparation of effector cells.*—Normal and *C. parvum*-stimulated peritoneal cells were harvested from groups of mice at various times after inoculation with M199 or 700 μg of CP. Mice were killed, the peritoneal cavity washed with 20 ml of M199 and the cells sedimented by 120 *g* for 5 min. These were then washed ×3 and resuspended to the required concentration in M199.

*Immunostimulating agent.*—*Corynebacterium parvum* (CP) was obtained from Wellcome Reagents Ltd, Hither Green, London, as a formalin-killed preparation (ULO1; preserved in 0·01% thiomersal) and dialysed against PBS before use, to remove toxic substances.

*Nylon-wool column fractionation.*—This method is described elsewhere (Julius *et al.*, 1973; Rees *et al.*, 1975). Briefly, 0·5 gm of nylon fibre was packed in a 5ml plastic syringe and saturated with M199 containing 5% FCS, for 30 min before loading with 2–5 × $10^7$ effector cells. The column was then incubated in a humidified atmosphere in 5% $CO_2$/95% air at 37°C for 40 min, and medium run through the column at a constant rate to obtain an eluted cell population. Column-retained cells were recovered by gentle teasing of the nylon fibres in M199.

*Glass adherence.*—One × $10^7$ effector cells suspended in M199 were incubated on glass Petri dishes at 37°C in a humidified atmosphere of 5% $CO_2$/95% air for 2 h. Non-adherent cells were removed by vigorous washing with M199 and adherent cells recovered by mechanical scraping. The cells were then washed ×3 and resuspended in M199.

*Carbonyl iron treatment.*—Four mg of carbonyl iron was added to $10^7$ effector cells in 1 ml of M199, and the mixture incubated at 37°C for 30 min. Carbonyl iron was sedimented on a magnet for 10 min at 4°C, the supernatant removed and the sedimentation repeated. The recovered cells were washed and adjusted to 5 × $10^7$/ml in M199 and used in experiments.

*Preparation and use of anti-Thy 1:2 antiserum.*—Anti-Thy 1:2 serum was prepared by immunization of AKR mice with 3 weekly injections of $10^7$ CBA thymocytes. The antiserum was collected one week after the 3rd immunization, and was shown to demonstrate selective complement dependent cytotoxicity for thymocytes from CBA and C57BL mice, but not AKR mice. This serum has also been shown to specifically abolish antitumour T-cell reactivity towards C57BL murine sarcomas (Kadhim & Rees, unpublished). Anti-Thy 1:2 treatment of effector cells was performed as follows: $10^7$ peritoneal cells were treated with an appropriate dilution of AKR anti-Thy 1:2 antiserum for 45 min at 4°C, centrifuged, and the supernatant removed; the cells were then incubated with guinea-pig complement for a further 30 min at 37°C, after which the remaining effector cells were washed ×3 in M199 before use.

*Chromium-51 release assay.*—Target cells ($10^6$ cells/ml) were labelled with 100μCi sodium chromate (Radio Chemical Centre, Amersham, Bucks) and incubated for 1 h at 37°C. The cells were then washed and resuspended in M199, and reincubated for a further hour. Following a second washing (×3), the targets were resuspended in M199 and adjusted to $10^5$ cells/ml. Target cells (0·1 ml) were mixed and incubated with effector cells (0·1 ml) in round-bottomed plastic microtest plates (NUNC-U-bottomed plates, Gibco Biocult, Paisley, Scotland). The E:T ratios ranged from 100:1 to 1:1. The plates were then incubated in a humidified atmosphere in 5% $CO_2$ in air at 37°C for 4 h, after which the plates were centrifuged at 200 *g* for 5 min and 0·1 ml of supernatant removed to another well. The plates were then allowed to dry, sealed with parafilm and the individual wells counted in a gamma spectrophotometer. The per cent $^{51}Cr$ release was calculated, following background subtraction, according to the formula:

$$\% \, ^{51}\text{Cr release} = \frac{(1/2 \text{ supernatant}) \times 2}{(1/2 \text{ supernatant}) + (\text{cells} + 1/2 \text{ supernatant})}$$

The per cent cytotoxicity was calculated by subtracting the % spontaneous $^{51}Cr$ release from targets incubated in medium alone (usually 5–10%) from the per cent $^{51}Cr$ release of the test.

*Adoptive transfer and direct-challenge experiments.*—Peritoneal cells from normal and CP-stimulated mice were collected, washed and adjusted to the required concentration. Equal volumes of effector cells and target cells were mixed, and 0·2 ml injected s.c. into groups of mice. The E:T ratio ranged from 1000:1 to 1:1; the target cells (lymphoma-EL4) were injected at $10^3$ cells/mouse, representing an inoculum of 10–20 $TD_{50}$ for this tumour.

Mice pre-treated with CP were challenged s.c. or i.p. with $10^3$ lymphoma cells and the development of tumours monitored over a 30-day period.

*Giemsa staining.*—Morphological identification of cytocentrifuged cell smears was performed. Slide preparations were air dried, fixed in methanol, and stained with 10% Giemsa for 10 min.

### RESULTS

### *In* vitro *cytotoxicity by* C. parvum-*stimulated effector cells*

Effector cells were harvested from the peritoneal cavity (PEC) of mice at different times after i.p. injection of 700 $\mu$g of CP, and assayed for cytotoxicity against K562 or EL4 targets in a 4h isotope-release assay. The results given in the Figure show the susceptibility of K562 and EL4 cells to CP-stimulated peritoneal effectors. A difference was found in the susceptibility of K562 and EL4 to PEC harvested on Days 2, 3, 4 and 7 after CP stimulation. The peak response to leukaemic K562 targets was seen on Day 2 and significant activity was also demonstrated on Days 3 and 4 (Figure). Consistent cytotoxicity to EL4 target cells could not be demonstrated on Day 2 (Tables I, II & III), but significant killing was apparent on Days 3 and 4 (not Day 7) after injection.

FIGURE.—Time course of reactivity of nylon-wool column-eluted *C. parvum*-stimulated effector cells (○), and nylon-wool column-retained effector cells to lymphoma EL4 targets (●). Peritoneal cells were harvested from groups of 3 mice, and pooled for fractionation.

Experiments were performed to com-

TABLE I.—*Cytotoxicity of adherent and non-adherent* C. parvum-*stimulated mouse peritoneal cells*

| Effector cells†: CP stimulated mouse PEC | % Cytotoxicity to target cells‡ K562 Exp. 1 | K562 Exp. 2 | EL4 Exp. 1 | EL4 Exp. 2 |
|---|---|---|---|---|
| Day 2 | | | | |
| Nylon-wool column-eluted | 21*** | 30*** | 0 | — |
| retained | 7 | 0 | 0 | — |
| Unfractionated | 23*** | 40*** | 1 | — |
| Glass adherent | 4 | 4 | −5 | — |
| non-adherent | 17* | 11** | 5 | — |
| Unfractionated | 23*** | 13** | 1 | — |
| Day 4 | | | | |
| Nylon-wool column-eluted | 2 | 8 | 1 | 4 |
| retained | −2 | 2 | 22** | 31** |
| Unfractionated | 1 | 3 | 13* | 11* |
| Glass adherent | −1 | — | 76*** | 30*** |
| non-adherent | 0 | — | 10 | 22** |
| Unfractionated | 3 | — | 11* | 13* |

† E:T ratio of 50:1.
‡ Significant cytotoxicity from *t* test: $*P < 0.05$; $**P < 0.01$; $***P < 0.001$.

pare the cytotoxic potential of CP-stimulated PEC, harvested 2 or 4 days after inoculation for both cell targets. PEC were fractionated on nylon-wool columns, and the column-eluted and retained cells were tested for cytotoxicity towards K562 and EL4 target cells. Similarly, glass-adherent and non-adherent effector cells were assayed for cytotoxicity, and the combined results of these studies are shown in Table I. The cytotoxicity of PEC harvested 2 days after CP stimulation to K562 targets was found preferentially in the nylon-wool-eluted and glass non-adherent fractions which displayed no apparent reactivity against EL4 lymphoma cells. In contrast, mouse PEC harvested 4 days after CP inoculation demonstrated preferential lysis of lymphoma EL4 cells, and most of the cytotoxicity was recovered from the nylon-wool-retained and glass-adherent fractions (Table I), though in one of two experiments, glass non-adherent effectors gave significant cytotoxicity to EL4 targets. These results show that effectors are non-adherent to glass and nylon wool, selectively cytotoxic to K562 targets, whereas cells adherent to glass and nylon wool are reactive to EL4 targets. Further evidence for 2 populations of effector cells in CP-stimulated PEC were found in studies of cell sensitivity to treatment with anti-Thy 1:2+complement, and carbonyl iron; these results are shown in Table II. Thus, cells harvested 2 days after PEC stimulation and reactive to K562 targets, were not affected by anti-Thy 1:2 + complement treatment, or carbonyl iron treatment. In contrast, cytotoxicity to both K562 and EL4 targets mediated by stimulated PEC harvested 4 days after (CP) inoculation, was removed by carbonyl-iron treatment but was unaffected by treatment with anti-Thy 1:2+complement (Table II). These results suggest that the effector cells present 2 days after CP stimulation, and mediating cytotoxicity to K562 are most probably NK cells, whereas the cells reactive to EL4 targets display the properties of macrophages.

To eliminate the possible involvement of polymorphonuclear leucocytes in cytotoxicity to K562 and EL4 targets, effector cells from 2 and 4 day CP-stimulated mouse PEC were fractionated on Ficoll–Triosil to yield an interface fraction mainly of mononuclear cells, and a pellet containing 65–75% polymorphonuclear leucocytes. When these cell fractions were assayed for cytotoxicity, the observed killing of both targets resided in the interface fraction; the polymorphonuclear leucocyte-enriched fraction failed to show any significant cytotoxicity (Table III).

Enrichment of cytotoxicity was found

TABLE II.—*Cytotoxicity of* C. parvum-*stimulated PEC fallowing removal of Thy 1 : 2 + lymphocytes and phagocytic cells*

| Effector cell† (mouse PEC) | % Cytotoxicity to target cells‡ | | | |
|---|---|---|---|---|
| | K562 | | EL4 | |
| | Exp. 1 | Exp. 2 | Exp. 1 | Exp. 2 |
| Day 2 | | | | |
| Anti-Thy 1:2+C | 20** | 12* | 5 | 1 |
| Carbonyl iron | 25** | 12* | −4 | −3 |
| Untreated | 25** | 15** | 7 | 0 |
| Day 4 | | | | |
| Anti-Thy 1:2+C | 18* | — | 10* | 13** |
| Carbonyl iron | 1 | — | −2 | 1 |
| Untreated | 14** | — | 10* | 21*** |

† E:T ratio of 50:1.
‡ Significant cytotoxicity from $t$ test: *$P<0{\cdot}05$; **$P<0{\cdot}01$; ***$P<0{\cdot}001$.

TABLE III.—*Lack of correlation between polymorphonuclear leucocyte content and cytotoxicity of CP-stimulated PEC*

| Ficoll–Triosil fraction: effector cells† (mouse PEC) | % PMN cells | % Cytotoxicity to target cells‡ | |
|---|---|---|---|
| | | K562 | EL4 |
| Day 2 | | | |
| Interface | 11 | 24** | 9* |
| Pellet | 72 | 3 | 3 |
| Unfractionated | 38 | 22** | 12** |
| Day 4 | | | |
| Interface | 10 | 20** | 36*** |
| Pellet | 67 | 2 | −4 |
| Unfractionated | 28 | 16** | 24** |

† E: T ratio of 50: 1.
‡ Significant cytotoxicity from $t$ test: * $P<0{\cdot}05$; **$P<0{\cdot}01$; ***$P<0{\cdot}001$.

in many of the separations and treatments performed. Failure to demonstrate an increase in cytotoxicity may relate to a loss of some of the effector cells during treatment. For example, murine NK cells have been shown to express Thy-1 antigen, and it is conceivable that some anti K562 effectors may have been destroyed by exposure to anti-Thy 1:2+complement (Koo *et al.*, 1980).

### In vivo *resistance to tumour transplantation*

Groups of mice were injected i.p. with 100 $\mu$g and 700 $\mu$g of CP, and 4 days later challenged s.c. or i.p. with $10^3$ EL4 cells. The incidence of tumours developing in the mice 30 days after inoculation is shown in Table IV. Ascites tumours developed in all control mice during the observation period (Table IV), and prior administration of CP by the i.p. route induced resistance to EL4 cells transplanted i.p. In contrast, no resistance was found in mice given CP by the i.p. route, and challenged s.c. (2 separate experiments) (Table IV).

TABLE IV.—C. parvum-*induced resistance to transplantation of lymphoma EL4 cells*

| Mice treated i.p. 4 days prior to tumour challenge ($\mu$g CP) | Route of tumour challenge† | Incidence of tumours 30 days after challenge | |
|---|---|---|---|
| | | Exp. 1 | Exp. 2 |
| 700 | i.p. | **0/4**‡ | **0/4** |
| 100 | i.p. | **0/4** | **0/4** |
| — | i.p. | 4/4 | 4/4 |
| 700 | s.c. | 4/4 | 4/4 |
| 100 | s.c. | 3/4 | 4/4 |
| — | s.c. | 4/4 | 4/4 |

† With $10^3$ viable lymphoma cells in 0·2 ml.
‡ Mice with tumours / Mice injected

The tumour resistance induced by prior inoculation with CP was further investigated in adoptive-transfer (WINN) assays. PEC were harvested at 2, 4 and 7 days after i.p. inoculation of mice with 700 $\mu$g of CP were mixed with $10^3$ viable lymphoma EL4 cells at PEC:tumour cell ratios of 1:1, 10:1, 100:1 or 1000:1, and each mixture inoculated s.c. into a group of mice. The incidence of tumours in these animals is shown in Table V. Control mice injected s.c. with $10^3$ EL4 lymphoma cells alone developed progressive lymphomas, whereas injection of PEC harvested 4 days after CP, in admixture with EL4 cells, caused a significant reduction in lymphoma incidence at E:T ratios of 1000:1, 100:1 and 10:1 (Table V). In contrast, PEC harvested 2 days after CP failed to inhibit EL4 lymphoma cells growth, and transfer of PEC harvested 7 days after CP was only effective in reducing the tumour incidence and size ($P<0·01$) at a 1000:1 ratio. The finding that adoptive transfer of only PEC harvested 4 days after CP inoculation prevented the development of EL4 lymphomas *in vivo* agrees with the results from the cytotoxicity tests. PEC from normal C57BL mice failed to influence the growth of EL4 cells in adoptive-transfer experiments (results not shown).

TABLE V.—*Adoptive transfer of Mouse PEC* 2, 4 *and* 7 *days after CP injection*

| E:T ratio | Incidence of tumours† — Time after CP injection (days) | | |
|---|---|---|---|
| | 2 | 4 | 7 |
| 1:1 | 6/8† | 5/8 | 8/8 |
| 10:1 | 6/8 | **3/8*** | 8/8 |
| 100:1 | 6/8 | **1/8** | 8/8 |
| 1000:1 | 5/8 | **1/8** | **4/8**** |
| 0:1 | 7/8 | 8/8 | 8/8 |

† Mice with tumours/mice inoculated 30 days after $10^3$ viable lymphoma cells in 0·2 ml were inoculated per mouse.
* $P<0·05$, ** $P<0·01$ — $t$ test.

To determine further which cell fraction was effective in preventing EL4 lymphoma growth, PEC were harvested from mice 4 days after inoculation with 700 $\mu$g of CP and the cells fractionated into nylon-wool-eluted and nylon-wool-retained populations. These fractions were separately mixed with $10^3$ lymphoma EL4 tumour cells at E:T ratios of 1:1, 10:1, 100:1 and 1000:1, and the mixtures inoculated s.c. into groups of mice. The incidence of tumours in these animals is shown in Table VI. The nylon-wool-eluted fraction

TABLE VI.—*Adoptive transfer of CP-stimulated mouse PEC*† *after nylon-wool column fractionation*

| | Incidence of tumours‡ | |
|---|---|---|
| E:T ratio | N.W. eluted cells | N.W. retained cells |
| 1:1 | 8/8 | 7/8* |
| 10:1 | 7/8 | **3/8*****  |
| 100:1 | 7/8 | **1/8** |
| 1000:1 | 7/8* | **0/8** |
| 0:1 | 8/8 | 8/8 |

† Harvested on Day 4 after CP treatment.

‡ 30 days after transfer. Significant reduction in incidence in bold.

Significant reduction in tumour size as determined by *t* test: *$P < 0.05$; ***$P < 0.001$.

of PEC showed no capacity to limit tumour growth; in contrast significant inhibition of tumour growth was found for column-retained cells at E:T ratios of 1000:1, 100:1 and 10:1. In addition, there was a significant delay in tumour appearance for mice inoculated with CP-stimulated nylon-wool-retained PEC transferred at 10:1 and 1:1 ratios ($P < 0.001$ and $P < 0.05$ respectively).

### DISCUSSION

The past decade has witnessed numerous attempts to suppress tumour growth by administering immune stimulants. Notable successes have been achieved, particularly following the intralesional injection of Bacillus Calmette Guèrin (BCG), which has caued the regression of large tumours in experimental animals (Zbar *et al.*, 1972; Baldwin & Pimm, 1973). Similarly, CP has also been shown to possess anti-tumour effects (Castro, 1974; Ojo, 1979; Milas & Scott, 1978). The results reported here indicated that CP-induced resistance to the growth of EL4 cells is restricted to the site of stimulation, and in this system at least, no systemic resistance is apparent. Our results agree with those reported by Castro (1974) who demonstrated local resistance to Meth A tumour cells in mice given i.p. CP. In contrast to this finding, Ojo (1979) has shown that CP administered i.p. induced systemic resistance to transplantation of the YAC lymphoma line. This tumour is NK-cell sensitive (Kiessling *et al.*, 1975; Herberman *et al.*, 1975; Ojo, 1979) while *in vivo*-derived EL4 cells are only weakly susceptible to NK cells (Herberman *et al.*, 1975); and the Meth A tumour has been reported to be killed by NC cells but insensitive to NK cells (Stutman *et al.*, 1978; Paige *et al.*, 1978). These observations suggest that CP enhances systematic NK reactivity, leading to effective *in vivo* growth inhibition of NK-sensitive targets. The results shown here indicate that local suppression of EL4 cells is not mediated by NK cells, but by cytotoxic macrophages.

Oehler *et al.* (1978) have reported enhanced PEC anti-tumour cytotoxicity in rats given i.p. CP, which was maintained for up to 5 weeks; however, the cytotoxicity was not fully characterized as being NK-cell or macrophage mediated. In the present study we have examined the induction/augmentation of cytotoxicity *in vivo* by CP, and shown that two distinct populations of cytotoxic effector cells are stimulated. The first, a non-adherent, non-phagocytic cell type showed selective killing of K562 cells and was present 2 days after *in vivo* administration of CP. Characterization studies suggest that these effector cells are NK cells. The second was glass adherent and phagocytic, and was present 4 days after CP administration, showing reactivity to EL4 target cells from an *in vivo* transplant line. The selective reactivity of these effector cells to different target lines suggests the expression of target receptor sites specific for different effector-cell populations.

Although the *in vivo* role of natural killer, and other naturally cytotoxic effector cell mechanisms, is not yet clear, there is evidence that NK cells may be of prime importance in immune surveillance against cancer cells (Kiessling & Wigzell, 1979) and may demonstrate *in vivo* cytotoxicity to blood-borne metastases (Hanna & Fidler, 1980). The results shown here would also suggest that tumour cells possessing an innate resistance to NK-mediated cytotoxicity may prove sensitive to phagocytic cells, which may constitute an important natural anti-tumour effector mechanism.

This work was supported by a grant from the Yorkshire Cancer Research Campaign. We wish to thank Mr A. Clegg, Mr A. Platts and Mrs D. Edey for their skilful technical assistance, and Mrs L. Rees and Mrs C. Mullan for typing the manuscript.

### REFERENCES

BALDWIN, R. & PIMM, M. (1973) BCG immunotherapy of a rat sarcoma. *Br. J. Cancer*, **28**, 281.

BALDWIN, R. W. & PIMM, M. (1978) BCG in tumour immunology. *Adv. Cancer Res.*, **28**, 91.

CASTRO, J. E. (1974) Antitumour effects of *Corynebacterium parvum* in mice. *Eur. J. Cancer*, **10**, 121.

DJEU, J. Y., HEINBAUGH, J. A., HOLDEN, H. T. & HERBERMAN, R. B. (1979*a*) Augmentation of mouse natural killer cell activity by interferon and interferon inducers. *J. Immunol.*, **122**, 175.

DJEU, J. Y., HEINBAUGH, J. A., HOLDEN, H. T. & HERBERMAN, R. B. (1979*b*) Role of macrophages in the augmentation of mouse natural killer cell activity by poly I:C and interferon. *J. Immunol.*, **122**, 182.

EINHORN, S., BLOMGREN, H. & STRANDER, H. (1978) Interferon and spontaneous cytotoxicity in man. I. Enhancement of the spontaneous cytotoxicity of peripheral lymphocytes by human leucocyte interferon. *Int. J. Cancer*, **22**, 405.

FLEXMAN, J. P. & SHELLAM, G. R. (1980) Factors affecting stimulation of natural cytotoxicity to a rat lymphoma by *Corynebacterium parvum*. *Br. J. Cancer*, **42**, 41.

GIDLUND, M., ORN, A., WIGZELL, H., SENIK, A. & GRESSER, I. (1978) Enhanced NK cell activity in mice injected with interferon and interferon inducers. *Nature*, **273**, 759.

HANNA, A. & FIDLER, I. J. (1980) Role of natural killer cells in the destruction of circulating tumour embolisms. *J. Natl Cancer Inst.*, **65**, 801.

HENNEY, C. S., TRACEY, D., DURDIK, J. M. & KEIMPEL, G. (1978) Natural killer cells *in vitro* and *in vivo*. *Am. J. Pathol.*, **93**, 459.

HERBERMAN, R. B., NUNN, M. E. & LAVRIN, D. H. (1975) Natural cytotoxic reactivity of mouse lymphoid cells against syngeneic and allogeneic tumours. I. Distribution of reactivity and specificity. *Int. J. Cancer*, **16**, 216.

JULIUS, M. H., SIMPSON, F. & HERZENBERG, L. A. (1973) A rapid method for the isolation of functional-thymus derived murine lymphocytes. *Eur. J. Immunol.*, **3**, 645.

KIESSLING, R., KLEIN, E. & WIGZELL, A. (1975) Natural killer cells in the mouse. I. Cytotoxic cell specificity for mouse Moloney leukaemia cell. Specificity and distribution according to genotype. *Eur. J. Immunol.*, **5**, 112.

KIESSLING, R. & WIGZELL, H. (1979) An analysis of the murine NK cell as to structure, function and biological relevance. *Immunol. Rev.*, **44**, 165.

KOO, G. C., JACOBSON, J. B., HAMMERLING, G. J. & HAMMERLING, U. (1980) Antigenic profile of murine natural killer cells. *J. Immunol.*, **125**, 1003.

MILAS, L. & SCOTT, M. T. (1978) Antitumour activity of *Corynebacterium parvum*. *Adv. Cancer Res.*, **26**, 257.

MOORE, M. & POTTER, M. R. (1980) Enhancement of human natural cell-mediated cytotoxicity by interferon. *Br. J. Cancer*, **41**, 378.

OEHLER, J. R., LINDSAY, L. R., NUNN, M. E., HOLDEN, H. T. & HERBERMAN, R. B. (1978) Natural cell-mediated cytotoxicity in rats. II. *In vivo* augmentation of NK-cell activity. *Int. J. Cancer*, **21**, 210.

OJO, E. (1979) Positive correlation between the level of Natural Killer cells and the *in vitro* resistance to syngeneic tumour transplants as influenced by various routes of administration of *Corynebacterium parvum*. *Cell. Immunol.*, **45**, 182.

PAIGE, C. J., FIGARELLA, E. F., CUTTICO, M. J., CAHAN, R. & STUTMAN, O. (1978) Natural cytotoxic cells against solid tumours in mice. II. Some characteristics of effector cells. *J. Immunol.*, **121**, 1827.

REES, R. C., BRAY, J., ROBINS, R. A. & BALDWIN, R. W. (1975) Subpopulation of multiparous rat lymph node cells cytotoxic for rat tumour cells and capable of suppressing cytotoxicity *in vitro*. *Int. J. Cancer*, **15**, 762.

SENIK, A., GRESSER, I., MAURY, C., GIDLUND, M., ORN, A. & WIGZELL, H. (1979) Enhancement by interferon of natural killer activity in mice. *Cell. Immunol.*, **44**, 186.

STUTMAN, O., PAIGE, C. I. & FIGARELLA, E. F. (1978) Natural cytotoxic cells against solid tumours in mice. I. Strain and age distribution and target cell susceptibility. *J. Immunol.*, **121**, 1819.

TRINCHIERI, G. & SANTOLI, D. (1978) Antiviral activity induced by culturing lymphocytes with tumour-derived or virus-transformed cells. Enhancement of human Natural Killer cell activity by interferon and antagonistic inhibition of susceptibility of target cells to lysis. *J. Exp. Med.*, **147**, 1314.

WOLFE, S. A., TRACEY, D. E. & HENNEY, C. S. (1976) Induction of "natural killer" cells by BCG. *Nature*, **262**, 584.

ZBAR, B., BERNSTEIN, I. D., BATLETT, G. L., HANNA, M. G. JR & RAPP, H. J. (1972) Immunotherapy of cancer: Regression of intradermal tumours and prevention of growth of lymph metastasis after intralesional injection of living *Mycobacterium bovis*. *J. Natl Cancer Inst.*, **49**, 119.